# Theoretical Antecedents of Standing at Work: An Experience Sampling Approach Using the Theory of Planned Behavior

**DOI:** 10.3934/publichealth.2016.4.682

**Published:** 2016-09-02

**Authors:** M. Renée Umstattd Meyer, Cindy Wu, Shana M. Walsh

**Affiliations:** 1 Department of Health, Human Performance, and Recreation, Robbins College of Health and Human Sciences, Baylor University, Waco, TX, USA; 2 Department of Management, Hankamer School of Business, Baylor University, Waco, TX, USA; 3 School of Education, Peru State College, Peru, NE, USA

**Keywords:** sedentary behavior, worksite, standing desks, sit-stand workstations, workplace, positive deviants

## Abstract

Time spent sitting has been associated with an increased risk of diabetes, cancer, obesity, and mental health impairments. However, 75% of Americans spend most of their days sitting, with work-sitting accounting for 63% of total daily sitting time. Little research examining theory-based antecedents of standing or sitting has been conducted. This lack of solid groundwork makes it difficult to design effective intervention strategies to decrease sitting behaviors. Using the Theory of Planned Behavior (TPB) as our theoretical lens to better understand factors related with beneficial standing behaviors already being practiced, we examined relationships between TPB constructs and time spent standing at work among “positive deviants” (those successful in behavior change). Experience sampling methodology (ESM), 4 times a day (midmorning, before lunch, afternoon, and before leaving work) for 5 consecutive workdays (Monday to Friday), was used to assess employees' standing time. TPB scales assessing attitude (α = 0.81–0.84), norms (α = 0.83), perceived behavioral control (α = 0.77), and intention (α = 0.78) were developed using recommended methods and collected once on the Friday before the ESM surveys started. ESM data are hierarchically nested, therefore we tested our hypotheses using multilevel structural equation modeling with Mplus. Hourly full-time university employees (n = 50; 70.6% female, 84.3% white, mean age = 44 (SD = 11), 88.2% in full-time staff positions) with sedentary occupation types (time at desk while working ≥6 hours/day) participated. A total of 871 daily surveys were completed. Only perceived behavioral control (β = 0.45, *p* < 0.05) was related with work-standing at the event-level (model fit: just fit); mediation through intention was not supported. This is the first study to examine theoretical antecedents of real-time work-standing in a naturalistic field setting among positive deviants. These relationships should be further examined, and behavioral intervention strategies should be guided by information obtained through this positive deviance approach to enhance perceived behavioral control, in addition to implementing environmental changes like installing standing desks.

## 1. Introduction

In addition to low levels of physical activity, emerging evidence suggests time spent being sedentary is also related with deleterious health outcomes [1],[2]. Sedentary behavior is a term used to characterize waking activities that require low levels of energy expenditure (i.e., less than 1.5 metabolic equivalents; METs) and a posture that involves sitting or reclining [3],[4]. Examples of common sedentary behaviors include prolonged sitting at a desk or computer, television viewing, sedentary commutes, and playing electronic games [3]. Researchers of large-scale studies have reported associations between time spent in sedentary behaviors and an increased risk of the development of chronic conditions including cardiovascular disease [5],[6], type II diabetes [7],[8], obesity [7], metabolic syndrome [9], colon cancer in men [10], and endometrial and ovarian cancers in women [11],[12]. In 2009, Katzmarzyk and colleagues published the results of a 12-year prospective cohort study that showed a progressively greater risk of mortality across greater levels of sitting [5].

Further, the relationships between sedentary behavior and health have been shown to be independent of, although related with, physical activity levels [1],[13], meaning a person who meets the recommended amount of physical activity specified in the U.S. national physical activity guidelines may still be at an elevated risk for negative health consequences if he or she also routinely engages in high levels of sedentary behavior. Given its public health importance, the Australian government and the Canadian Society for Exercise Physiology have issued evidence-based national sedentary guidelines that exist in addition to their country's physical activity guidelines [14],[15]. The U.S. has not yet issued sedentary guidelines, though some discussion surrounding their development has begun [13]. Sedentary behavior guidelines may be particularly important in the U.S., where researchers have found adults spend an average of 8.44 hours waking hours per day in sedentary behavior [16].

Among many U.S. adults, work is a large source of sedentary behavior. According to a study published in 2011, an escalating number of U.S. jobs require low levels of energy expenditure, with only about 20% of U.S. jobs requiring even moderate intensity physical activity compared to 50% of jobs in the 1960s [17]. Researchers in Australia and the U.K. have reported between 71%–82% of office workers' time at work being spent in sedentary time [18]–[20]. Evidence from the U.S. has shown that on work days, sitting at work accounts for 63% of total daily sitting time [21]. Findings also suggest that persons with sedentary occupations do not compensate by engaging in lower levels of sedentary behavior in their leisure time, and that sitting in one segment of life is related with sitting in other segments of life [22],[23]. This further emphasizes the need for efforts to reduce sedentary behaviors, with particular attention being paid to the workplace as a venue for sedentary reduction efforts.

Popular worksite interventions to reduce sedentary time include the use of standing or sit-stand workstations (e.g., height-adjustable desks that can be worked from in either sitting or standing positions), treadmill desks, and pedal desks. Preliminary evidence from a systematic review and meta-analysis suggests all three have the capacity to reduce sedentary time at work without interfering with employee productivity [24]. Standing desks or sit-stand workstations may be particularly feasible given their comparatively low cost and the low risk associated with their use. Standings desks, given their purpose, promote increased levels of standing throughout the workday. Despite evidence suggesting similarly low levels of energy expenditure, standing represents a physiologically state different from sitting [25] by engaging large muscle mass in the lower extremities that remain inactive in the seated or lying positions [26]. Health benefits associated with standing include reduced musculoskeletal pain [27], slightly increased energy expenditure [28], and a reduced risk of premature mortality [5],[6]. Additionally, standing desks have been reported to have high levels of usability and acceptability in office settings [29], making them a viable option for sedentary reduction efforts in the workplace.

Given the negative health consequences of time spent being sedentary, the high levels of sitting associated with U.S. jobs, and the evidence supporting the use of sit-stand workstations, conducting research to understand this relatively new behavior of standing at work is warranted. While environmental changes to support reduced sitting time at work (e.g., supplying sit-stand desks/work stations) seem to be essential based on previous literature, evidence also suggests that multi-component approaches that include behavioral intervention strategies in addition to environmental changes produce the strongest effects [30]. However, limited work has been done examining which behavioral intervention strategies are the most effective. Applying theory to the understanding and explaining of standing or sitting at work may be particularly useful in this area, and could be used to lay the groundwork in designing more effective behavioral components of interventions to reduce sedentary time at work. Researchers have previously reported behavior change interventions based in theory to be more effective than their atheoretical counterparts [31],[32], though this is in contrast with results from a recent meta-analysis that indicated the application of theory to an intervention made intervention effectiveness no more likely [33]. However, the authors of this meta-analysis only had sufficient data to examine the Social Cognitive Theory and the Transtheoretical Model, reported that few studies in their sample applied theory extensively, and concluded with recommendations for continued application of health behavior theories to understand health behaviors. Despite continued recommendations and support to apply theory to behavior change interventions, few worksite sedentary reduction interventions, if any, have applied theory to date.

The Theory of Planned Behavior (TPB) is one behavioral theory that has shown strong predictive value across a variety of health behaviors and has also been particularly useful in explaining intention and behaviors similar to standing, such as sedentary behaviors (e.g, sitting time, television watching, playing video games, computer use, reading, sitting for work/school, leisure sitting, transportation sitting, etc…), physical inactivity, and physical activity [34]–[40]. The TPB is an extension of the Theory of Reasoned Action (TRA), which was originally developed to explain volitional behaviors through three constructs: attitude, subjective norm, and intention [41],[42]. Theoretically, based on TRA, intention to stand (versus sit) while working at a desk is directly related with an individual's standing behavior, and intention is influenced by attitude (perceived benefits and costs of standing while working at a desk, the value placed on these, and positive or negative evaluation of how the behavior was performed) and subjective norms (perceived approval, support, or lack thereof from important others regarding standing at a desk to work). To better account for behaviors that are not fully volitional, the TRA was revised as the TPB through the addition of the construct perceived behavioral control (the amount of control an individual perceives he/she has to stand while working at a desk), where perceived behavioral control is both indirectly related with behavior through intention and directly related with behavior [41],[42].

Empirically, to date, intention to be sedentary has been supported as a direct determinant of sedentary or inactive behaviors [34]–[36], where intention has been influenced by attitude [34],[35], subjective norm [34]–[37], and perceived behavioral control [35]–[37], and perceived behavioral control has also been directly related with the behavior [35]–[37]. Interestingly to note, some evidence has also suggested a direct relationship between attitude and sedentary behavior [34],[38]. In 2009, Rhodes and Dean found that intention to perform four leisure sedentary behaviors (television viewing, computer use, reading/music, and socializing) was consistently related with sedentary behavior and that attitude was associated with all sedentary behaviors through intention and subjective norm was related with intention in the expected direction. However, the theoretically suggested relationships for perceived behavioral control with intention and sedentary behaviors were not supported [34]. Other research supports an inverse relationship between attitude for physical activity and lower levels of sedentary behavior in adults with advanced brain cancer [38]. And, most recently, researchers found TPB constructs to explain between 9–58% of the variance in intention and 8–43% of the variance in four different classifications of sedentary behaviors of Canadian adults (weekday work/school, weekday leisure/recreation, weekend work/school, and weekend leisure/recreation; leisure/recreation reflected behaviors with more volitional control and work/school those behaviors with less volitional control) [35]. Intention was significantly and positively related with all four sedentary behavior types, and attitude and subjective norm were related with intentions for two sedentary behaviors and two of the sedentary behaviors directly. Greater perceived behavioral control was significantly related with less time spent in weekday work/school sedentary behavior and lower intentions to spend time in weekday work/school sedentary behavior.

Understanding the characteristics, behaviors, and strategies related with natural engagement in a behavior can help guide more effective intervention development, especially in populations or for behaviors that are not very well understood, like sitting and standing behaviors. Individuals in a community that have been successful in an area of interest (e.g., behavior adoption/maintenance), as compared to their peers, are referred to as “positive deviants”. Marsh and colleagues [43] describe three distinct steps in using positive deviance to help resolve challenges: (1) positive deviant identification; (2) study and observe the positive deviants; and (3) design behavior change intervention strategies using the information gained from step #2. One premise and strength of a positive deviance approach is that interventions are designed based on how people within a community are already practicing a behavior of interest given their available resources, thus increasing the likelihood of adoption and sustainability [43]. Although, Marsh and colleagues recommend qualitative approaches to understand positive deviants, researchers have also applied and recommended the use of pre-existing/archived quantitative data to guide a positive deviance approach [44]. In this approach positive deviants are identified, and previously collected data from these positive deviants are used to assess the area of interest (e.g., health behavior, health problem) and identify characteristics of the positive deviants, situational/contextual factors potentially relevant to the area of interest, and normative patterns for identified situational factors. In working with datasets in this fashion, it is essential to ensure that data exist not only on the health outcome of interest, but also on associated health behaviors and related antecedents for these behaviors such as attitudes, beliefs, and perceptions [44]. Given the recommendations provided by Walker and colleagues [44] for using existing datasets, it is plausible that designing and using surveys to collect new data could provide useful and potentially more complete information, compared with accessing existing data, when resources are limited or time does not allow for more intensive qualitative approaches. Collecting new data from identified positive deviants would provide useful information to help in understanding healthy/positive behaviors and guide intervention development, since surveys could be designed to better ensure that the resulting dataset includes information about positive deviants' behaviors, situations, characteristics, and related antecedents.

In light of the need to better understand how to reduce sitting time in our adult population, and current evidence supporting sedentary behaviors as largely volitional and therefore well-aligned for TPB application [34],[36],[41], the purpose of this study was to examine the relationships between TPB constructs and time spent standing at work among a sample of positive deviants. Specifically, we sought to examine the relationships between work standing behaviors and TPB constructs among employees that were already using standing desks in their work environment to answer the research question: Are TPB constructs related with standing behaviors in a sample of employees who are already using standing desks at work? Although findings from current sedentary behavior research are not fully consistent across studies, we hypothesized that the theoretically posited relationships between TPB constructs and standing behavior would be observed in the present study. Specifically, more positive attitudes, greater social norms, and greater perceived behavioral control will be related to greater intention, and greater intention and perceived behavioral control will both be related to greater time spent standing during work.

## 2. Materials and Methods

### 2.1. Participants and Recruitment

After obtaining Institutional Review Board approval, full-time male and female employees with sedentary occupation types (i.e., ≥6 hours per day spent at a desk) were recruited from a university and the surrounding central Texas community to participate as positive deviants in this study. In addition to having a full-time sedentary job, participants were also required to be between the ages of 20 and 65, be ambulatory, not have a medical condition that contraindicated standing, and not be pregnant. Participants with previously established standing workstations were invited to participate (no participants were provided with standing workstations for use in this study). Recruitment took place via announcements posted to a staff and faculty webpage, mass emails sent to university staff and faculty, snowball searches with study participants, and personal email and telephone communication.

### 2.2. Procedures

Persons interested in participating in the study were invited to an in-person meeting in a campus laboratory with a study investigator. Upon providing informed consent, participants completed surveys designed in Qualtrics on a computer in the laboratory that included items assessing demographic characteristics and theoretical constructs. During their visit, participants were also introduced to experience sampling methodology (ESM). ESM allows the participants to recall their standing time at shorter time intervals and thus capture a person's representation of an experience as it is occurring [45]. Specifically, we utilized the interval-contingent ESM (participants recording their experiences at equal intervals) by which “a recollection of events transpiring in the preceding interval (e.g., day or week) will provide the researcher with data addressing his or her questions” [46].

To use ESM in this study, participants were asked to complete five brief Qualtrics surveys during the week following their initial survey completion (in the morning at the beginning of the workday, midmorning between 9:30 and 10:30, before lunch, afternoon between 2:00 and 3:00, and before leaving work) for five consecutive workdays (Monday to Friday). In each of these surveys, participants indicated the amount of time they spent sitting, standing, or other. These surveys were sent automatically from Qualtrics via email to each participant at the same scheduled times each day over their one week of participation. As an incentive for participation, participants (n = 50) were compensated with one of three incentives valued at $5 or less (e.g., free beverage card at a national coffee shop). Data collection took place between April and July of 2015. The morning survey delivered at the beginning of the day was used as a baseline and therefore was not included in our data analysis.

### 2.3. Measures

Sociodemographic characteristics and health measures. Selected survey items from the Behavioral Risk Factor Surveillance Survey were asked as part of the online Qualtrics survey to ascertain age, sex, race, ethnicity, marital status, number of children, and highest level of education attained. Height and weight were measured objectively using a digital scale and stadiometer (Detecto PD300DHR, Detecto Scale, MO), and data were used to calculate body mass index (BMI) using the following formula: weight (kg)/height (meters^2^) [47].

Theoretical constructs. Survey items assessing TPB constructs (attitude, subjective norm, perceived behavioral control, and intention) were developed by the researchers based on the TPB Construction Questionnaire [48], and a study using the TPB to measure leisure-time sedentary behaviors [34]. To best ensure that TPB constructs captured all perceptions surrounding standing behaviors at work, items referencing both standing and sitting behaviors were included for each TPB construct since sitting is the primary alternative to standing for deskwork.

Attitude. Eight items were used to assess attitude in this study, with four items asking about sitting for six or more hours per day while at work, and four items asking about standing while working at a desk during the workday. Items were anchored on a 7-point scale of bipolar adjectives that included: harmful/beneficial; unpleasant/pleasant, unenjoyable/enjoyable, and unwise/wise (e.g., sitting for 6 hours or more per day while at work is harmful/beneficial). Exploratory factor analysis (EFA) showed that two factors emerged from these attitude items (4 items pertaining to attitude toward standing, and 4 items pertaining to attitude toward sitting), with rotated factor loadings all greater than 0.60 using varimax rotation. Consequently, the four items worded for sitting were combined into the attitude toward sitting scale (Cronbach's α = 0.84), whereas the four items worded for standing were combined to form the attitude towards standing scale (Cronbach's α = 0.81).

Subjective norm. Four items were used to assess subjective norm, with two items referring to increasing standing at a desk while at work and two items referring to reducing sitting at a desk while at work. All items were written with “people who are important to me” as the stem (e.g., people who are important to me would approve of me standing at my desk at work). These items addressed injunctive norms (e.g., beliefs people have about whether those who are important to them would approve or disapprove of their performing the behavior). All items were anchored on a 7-point scale with “disagree” and “agree” as endpoints. EFA showed that one factor emerged from these four items with factor loadings all greater than 0.60, thus they were combined and the mean score was used to represent the level of perceived subjective norm. Cronbach's α was 0.83.

Perceived behavioral control. Perceived behavioral control was measured with two items on a 7-point Like scale with “disagree” and “agree” as endpoints. One item addressed sitting and one item addressed standing. Both asked about feelings of control over the behavior (e.g., I have control over how many hours per day I sit while at work). EFA showed that one factor emerged from these two items with factor loadings all greater than .60, thus they were combined and the mean score was used to represent the level of perceived behavioral control. Cronbach's α was 0.77.

Intention. Four items were used to measure intention, each anchored on a 7-point Likert scale with “disagree” and “agree” as endpoints. Two items addressed reducing time spent sitting at work while two items addressed increasing standing at work (e.g., I plan to reduce the number of hours per day I spend sitting at my desk while at work). EFA showed that these four items loaded on the same factor also with factor loadings all greater than 0.60, therefore they were combined and the mean score was used to represent the level of intention. Cronbach's α was 0.78.

Work desk standing behaviors. Time spent standing was measured via ESM, where participants were emailed surveys automatically at the same five time points throughout the day (morning, mid-morning, before lunch, mid-afternoon, and end of workday) for five consecutive workdays (Monday to Friday) using Qualtrics. If they were not able to complete the survey during the suggested period, participants were advised not to complete the survey. In this way, we avoided participants waiting until the end of the day to complete all surveys, which would have not been consistent with ESM procedures designed to capture experience as it is happening. Starting with the mid-morning survey, participants were asked the question “Please report what percent of time you spent *standing* since you completed the last survey.” Percentages of time spent standing reported by participants starting with the mid-morning survey were used in final analyses. 871 daily surveys were completed, resulting in a completion rate of 87%. Thirty-eight of these 871 daily surveys were not completed within the allotted time window, rendering less than 5% latency rate. Although not within the allotted survey time window, these 38 surveys were completed before the next survey time in the day. Consequently, we retained all 871 daily surveys in our analysis.

### 2.4. Analyses

Data analyses were conducted using Mplus 7.4 [49]. Descriptive statistics were computed for sociodemographic variables and used to describe the sample, and bivariate correlations between variables were examined. Because ESM data are hierarchically nested (e.g., multiple data points over time for each participant), multilevel structural equation modeling was used to examine the relationships between TPB constructs and standing behaviors. We controlled for participants' age and sex in the analysis given previous evidence supporting that both sex and age are related with sedentary behavior (e.g., females are more sedentary than males and sedentary behavior increases with increasing age [38],[50]) and behaviors similar to sedentary behavior like physical activity (e.g., males are more active than females and physical activity declines as one ages [51]–[54]).

Prior to estimating models, we conducted diagnostic assessments. Specifically, we estimated the intra-class correlations (ICC(1)) of standing behavior to determine if there was sufficient variance at both the within- and between-persons levels [40]. ICC(1)s as low as .05 indicate level-2 effects that warrant further examination [55]. Our data suggested that ICC(1) was sufficiently high to justify person-level effects (0.19 at the 0.01 level). The use of multilevel modeling to examine the between-person level antecedents (i.e., TPB constructs) while controlling for within-person variance was supported.

## 3. Results

### 3.1. Sociodemographic Characteristics

Table 1 displays descriptive characteristics of the study sample. Participants were mostly middle-age, white, women, and in non-academic staff positions (i.e., not “faculty” or academic staff).

### 3.2. Bivariate Correlations

Bivariate correlations between study variables are presented in Table 2. Participants reported standing for an average of 57.78% of the time since they completed the previous survey. Although percentage of time spent standing was not directly related with any of the variables of interest, perceived behavioral control was significantly related with intention to stand in bivariate analyses.

**Table 1.** Study Sample Characteristics (n = 50).

Variables	Mean/Count *(range)*	SD/%
Sex		
Female	36	70.6%
Male	14	27.5%
Age	44.14	10.95
Race		
African American	1	2.0%
Asian	1	2.0%
Hispanic	4	7.8%
White non-Hispanic	43	84.3%
Other	1	2%
BMI	26.68 *(19.13–41.22)*	4.82
Number of children	1.66 *(0–4)*	1.17
Marital Status		
Married/partnered	41	80.4%
Non-married	9	17.7%
Educational Attainment		
H.S. / GED	1	2.0%
Some college	9	17.6%
4-yr college grad	20	39.2%
Graduate degree	20	39.2%
Job category		
Faculty	5	9.8%
Staff	45	88.2%
Hours of work/day ^a^	8.13	1.16
Hours sitting at work/day ^b^	3.39	1.87

*Note:* ^a^ Measured with one question: On average, how many hours do you work per day? ^b^ Measured with one question: On average, how many hours per day do you spend sitting while at work?; BMI: body mass index.

**Table 2.** Person-level Descriptive Statistics and Correlations.

	Mean	SD	1	2	3	4	5	6	7	8
1. Age	44.14	10.95	-							
2. Sex ^a^	1.28	0.45	-0.19	-						
3. Attitude toward sitting	2.57	1.28	0.03	-0.23	(0.84)					
4. Attitude toward standing	6.24	0.82	0.09	0.09	0.09	(0.81)				
5. Subjective norms	6.16	1.13	0.13	0.08	-0.44**	0.27	(0.83)			
6. Behavioral control	6.14	1.04	0.37**	-0.15	0.14	0.18	-0.08	(0.77)		
7. Intention	5.97	1.25	-0.07	-0.11	0.10	0.36*	0.27	0.55**	(0.78)	
8. Standing behavior ^b^	57.78	18.47	0.11	-0.02	-0.09	0.15	0.18	0.23	0.11	-

*Notes:* n = 50. Scale reliabilities are shown in parentheses along the diagonal. Behavioral control = perceived behavioral control; a: sex: 1 = female, 2 = male; b: percentage of time spent standing since last survey completion. *p < 0.05; **p < 0.01.

### 3.3. Multilevel Modeling

**Figure 1.**
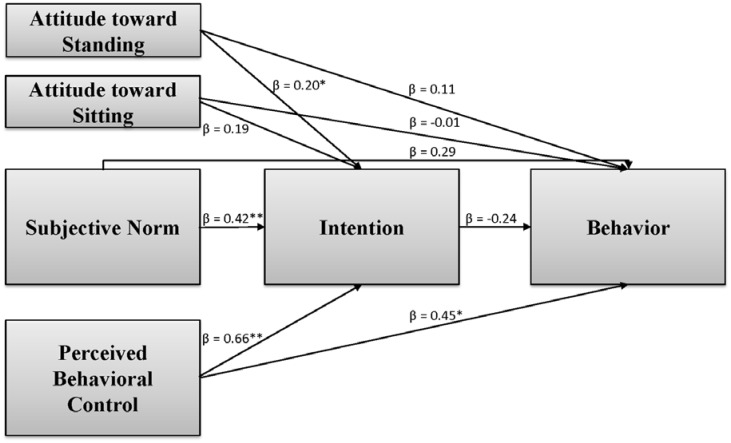
Multilevel path analysis based on current standers (n = 50). Event-level n = 871. Standardized path coefficients reported, controlling for age and sex, neither of which was related with standing behavior or intention except that age was negatively related with intention to stand (β = -0.41, *p* < 0.01). * *p* < 0.05; ** *p* < 0.01.

We then examined the relationships between TPB constructs and standing behavior among current standers while controlling for age and sex. A total of 871 event-level surveys completed by 50 participants (completion rate = 87%), were included in this analysis. Because of the exploratory nature of the study, we estimated a saturated model (i.e., every possible path was estimated) where the data just fit the model. A saturated model is particularly suitable when “no existing theory or a priori hypothesis can be used to develop further parameter constraints rendering a model under consideration with positive degrees of freedom” (p. 1055) [56]. Although not without critique, conventionally saturated models yield “perfect model fit”, resulting in χ^2^(0) = 0, CFI and TLI = 1, and RMSEA and RMR = 0 [56]. Results of this model are shown in Figure 1, demonstrating that only perceived behavioral control (β = 0.45, *p* < 0.05) was directly related with time spent standing; although attitude toward standing (β = 0.20, *p* < 0.05), subjective norm (β = 0.42, *p* < 0.01), and perceived behavioral control (β = 0.66, *p* < 0.01) were related with intention.

## 4. Discussion

Accumulating evidence supports the detrimental health impacts of sedentary behavior and the health benefits of standing. Many U.S. jobs, however, require desk work that is commonly conducted from a seated position. With the emergence of standing desk variants and the positive findings supporting their use, research objectives designed to better understand standing behaviors at work among employees with jobs that require desk work are warranted. The objective of this study was to examine relationships between TPB constructs and sitting and standing behaviors of employees who were already using standing desks. Primary findings suggest the usefulness of the TPB in understanding standing behaviors of employees with standing desks.

In this study, and similar to the procedures of others [34],[35],[38], an exploratory approach to the application of the TPB was tested, where a saturated model was examined. Although a large body of literature across a variety of health behaviors supports the predictive value of the TPB in its traditional form [40],[57], standing at work represents a new health behavior that has not yet been examined in relation to these theoretical constructs. By testing a saturated model, the traditional model is still examined, and relationships that may have the potential to enrich our understanding of the behavior may also be revealed.

Fifty employees with current standing workstations were enrolled in this study as a means of examining the “positive deviants”, or the persons already participating in the positive health behavior of standing at work [43]. Because this group of participants chose to engage in a healthy behavior of their own accord, and can be considered successful and “early adopters”, understanding behavioral and psycho-social factors including TPB constructs that are related with their behavior can be particularly insightful. Findings from this model revealed significant positive relationships between intention and attitude toward standing, subjective norm, and perceived behavioral control. This is consistent with previous studies supporting these theoretically posited relationships [34]–[37]. Results also align with previous related work supporting the theoretically-posited direct relationship between perceived behavioral control and time spent standing [35]–[37]. However, in our sample intention was not related with time spent standing, and therefore theoretically posited mediation relationships could also not be examined. Although this does not align theoretically with the TPB, two other studies examining sedentary behavior have also failed to find a relationship between intention and sedentary behavior [37],[38]. One potential explanation of this could lie in employees' perceptions of how volitional standing at work actually is. Given that there are a number of barriers seemingly out of one's control at work (e.g., demands of job, meetings, environmental barriers, etc.), it could be that while intention is still related with attitude, subjective norms, and perceived behavioral control, it is not related with actual time spent standing. Rather, actual time spent standing is most influenced by an individual's perceptions of whether he/she has control over standing at work, Or, another way of thinking about this would be the degree to which an individual believes that standing at work is easy or difficult [41]. Although standing at work seems to be similar in nature to other sedentary behaviors, especially weekday work/school sedentary behaviors [35], this is the first study we are aware of that has specifically examined TPB in relation to time spent standing while working at a desk. And while the TPB is “a theory designed to predict and explain human behavior in specific contexts” (p. 181) [58], as was done in our study by focusing specifically on standing behaviors at work, this also means that previous sedentary-focused research is similar to, although distinct from the specific behavior we examined [58]. One other potential explanation could lie in the operationalization of the construct. We asked specifically about intention for “reducing sitting time” and “increasing standing time”. Given that these individuals were all already engaged in some level of standing at work, our wording possibly could have confused respondents. Had we asked about “continuing to stand”, we may have had different results.

Descriptive analyses from this study revealed that, on average, employees who had sit-stand workstations spent 3.39 hours seated per workday. The average percentage of time these positive deviants spent standing during each survey interval (about 2 hours) was 57.78%. Although not surprising, these data support the efficacy of using standing desks to reduce sedentary time among employees with sedentary job types. Standing desks, specifically height-adjustable sit-stand workstations, are recommended as environmental-change components of sedentary reduction interventions [30],[59]–[61]. However, recent findings from a meta-analysis support and recommend multi-component approaches as potentially more effective when compared to approaches including environmental changes alone (e.g., adding behavioral change components to standing desk interventions: motivational interviewing; information on behavioral consequences related with sedentary time; self-regulatory strategies including goal setting, action planning, self-monitoring prompts; cues to action; and social norms) [30]. It should also be noted though that a recent Cochrane Review found only very low and low quality evidence in the literature supporting worksite standing interventions [62]. The authors of the review suggest more rigorous study designs and methodology be applied to examine these interventions. In addition to more rigorous designs, we also suggest incorporating the findings from research that examine theoretical constructs using a positive deviance approach, as interventions designed using information gained from “successful” community members are supported as more adoptable and sustainable [43]. In our model with a sample of positive deviants, perceived behavioral control was significantly related with both intention and behavior, and subjective norm and attitude toward standing were related with intention to stand. Interventions and efforts to promote standing that incorporate components addressing norms and perceived behavioral control may be especially successful and align with Chu and colleagues recent recommendations [30].

This study represents the first application of the TPB to examine the behavior of standing at work in a sample of positive deviant employees. The results add to and build upon a developing body of literature applying theory to sedentary behavior [34]–[38]. The primary findings, which support the use of the TPB in explaining standing, can be used to support efforts to promote standing at work and help explain this behavior. Despite study strengths, there are several limitations worth noting. First, our sample of current standers was potentially limited with only 50 participants. All were from Central Texas, and the majority were white, female, overweight, and held full-time non-academic staff positions in a university setting. It is possible that a less homogenous sample would yield different results. The behavior of interest in this study, standing while working, was measured via self-report. Although there is no direct evidence suggesting the accuracy of self-report percentage of time spent standing, recent research shows that self-report measures of sitting time in the domain-specific diary (assessed by asking participants to report time spent sitting each day in areas such as work and commuting) did not differ significantly from accelerometer-determined sedentary time [63],[64]. This suggests that participants can recall their time spent in a certain posture (standing or sitting) with reasonable accuracy even when it was recalled on the once-a-day basis. Experience sampling methodology (ESM) utilized in this study allowed the participants to recall their standing time at even shorter time intervals (in our case, 4 times a day through the work hours, instead of once a day, single question, at the end of the day). Participants were asked to report their standing and sitting behaviors over the previous ∼2 hours. Additionally, our sample was comprised of adults with sedentary job types (defined in this study as ≥6 hours per day of desk work). Because participants were asked to report standing and sitting in the previous ∼2 hours, and they were most likely in their offices working at their desks during that time, we believe that they could accurately estimate their standing and sitting behaviors over that time. Taken together, although there is no specific evidence supporting the ability of participants to accurately report standing and sitting percentage of time, we believe using EMS is the best possible subjective measurement of standing and sitting behavior. Still, we encourage future research to employ an objective measure of standing time, such as the activPAL, to strengthen the findings.

Because the TPB had not previously been used to examine standing behaviors, all survey items were developed for this study using guidance from the TPB Construction Questionnaire [48]. In an effort to capture the behavior holistically, survey items were worded to address both sitting and standing for each construct for individuals working at a desk. Although internal consistency across items was acceptable and the EFA revealed acceptable fit, this is a potential weakness of the study. Additional research is needed to validate and potentially build upon the survey items used in this study. For example, survey items addressing TPB constructs related to sitting referred to a specific timeframe (≥6 hours per day), whereas items related to standing did not refer to a timeframe. Because there is no current recommendation for an amount of time spent standing as related to health benefits, a timeframe was not included with those survey items. However, this inconsistency is also a potential limitation of the measures of the study. Lastly, survey items measuring subjective norm only assessed injunctive norms and did not refer to specific groups (e.g., co-workers, family members, friends), and instead only were asked in regards to “people who are important to me”. It is possible that referencing specific groups could alter respondents' perceptions of their social norms. Future research should examine social norms as they pertain to specific referent groups to better understand if there are specific groups that are more relevant for standing behavior. Future work should also include items to assess both injunctive and descriptive norms.

We also suggest that future research further explore within-person predictors for standing. ICC(1) of 0.19 in standing behavior represented sizable between-person level variance that is noticeably above the suggested threshold of 0.05 [55] and therefore justified the examination of the between-level antecedents. However, 81% of the variance in standing was still attributed to within-person factors. This level of within-person variation does not seem uncommon given that previous research also reported about half of the variance in self-reported sedentary behavior and only one quarter of the variance in monitored sedentary behavior were at the between-person level [64]. An episodic approach that examines the within-person level of events (such as meetings, student advising appointments, higher level of required mental concentration, one's energy level at the time, time of the day, etc.) that facilitate or hinder standing while working warrants promise for such discovery. In addition, future research should also continue to explore theoretical determinants of standing, using the TPB and other health behavior theories, in an effort to better understand the behavior and therefore more effectively promote it. Lastly, previous research has reported disparities between women and men in their sitting behavior [23], and therefore we encourage researchers to emphasize women as a priority population in public health efforts.

## 5. Conclusion

Overall, our results provide support for the use of the TPB to help understand work-related standing behaviors for employees with sedentary job types. The results also suggest that having a standing desk is related to both standing and less sedentary behavior as compared to national averages. Increasing perceived behavioral control, attitudes towards, and subjective norms for standing may also increase their intention to stand and the amount of time employees spend standing. Given the negative health consequences of sitting and the multitude of jobs that require people to sit in today's society, a better understanding of standing at work can be useful in guiding health promotion initiatives to encourage standing as a healthy alternative to sitting at work.
